# Supplementary material for: Cytotoxicity of Ficus Crocata Extract on Cervical Cancer Cells and Protective Effect against Hydrogen Peroxide-Induced Oxidative Stress in HaCaT Non-Tumor Cells
Source: Plants (Basel). 2021 Jan 19;10(1):183. doi: 10.3390/plants10010183 (PMC7835743; doi:10.3390/plants10010183)
Supplement: Supplementary file 1 [file plants-10-00183-s001.zip › Figure S1.pdf]

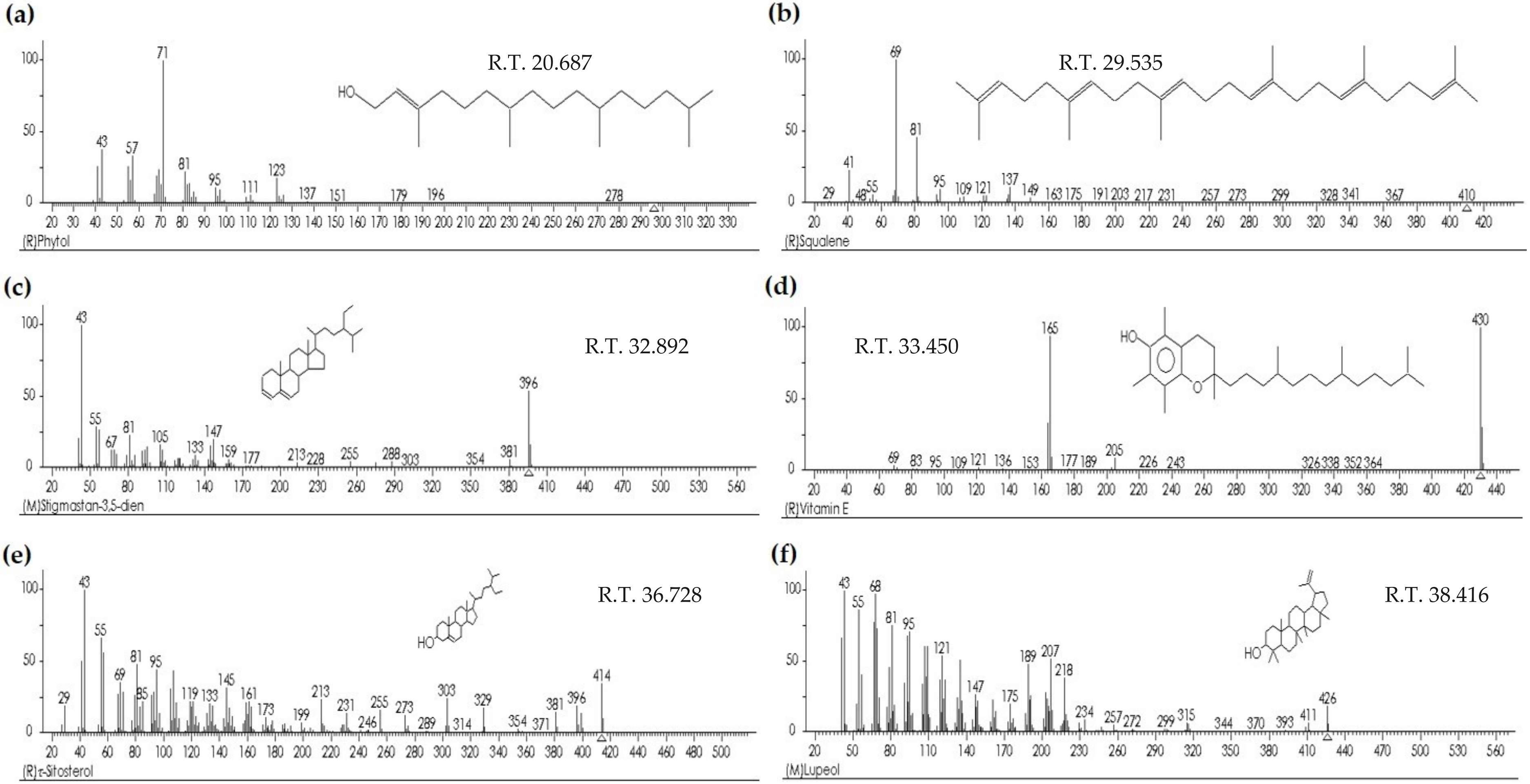

Supplementary figure 1. GC-MS analysis of acetone extract of *Ficus crocata (Miq.) Mart. ex Miq.* leaves. Chromatogram and structure of the compounds identified in Ace-EFc,. (a) Phytol; (b) squalene; (c) Stigmastan-3,5-dien; (d) alpha-tocopherol; (e) β-sitosterol; (f) lupeol. R.T: retention time.
