# Supplementary material for: Cytotoxicity of Ficus Crocata Extract on Cervical Cancer Cells and Protective Effect against Hydrogen Peroxide-Induced Oxidative Stress in HaCaT Non-Tumor Cells
Source: Plants (Basel). 2021 Jan 19;10(1):183. doi: 10.3390/plants10010183 (PMC7835743; doi:10.3390/plants10010183)
Supplement: Supplementary file 1 [file plants-10-00183-s001.zip › Figure S2.pdf]

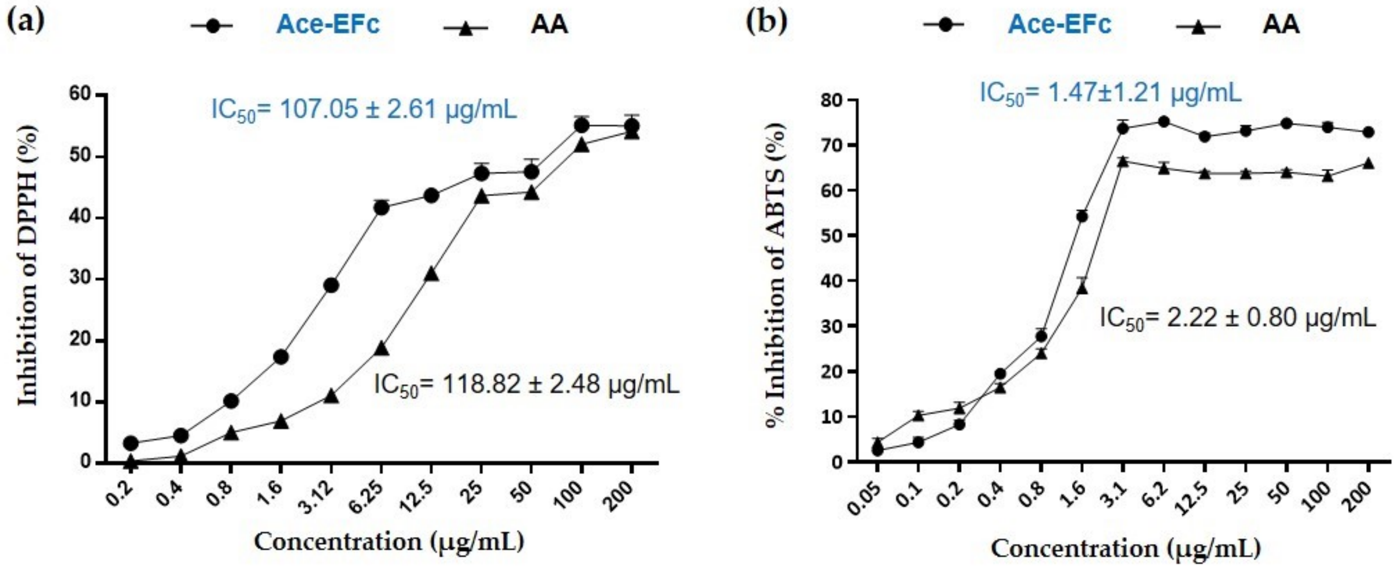

Supplementary figure 2. Antiradical activity of Ace-EFc. (a) DPPH and (b) ABTS free radical scavenging activity of acetone extract of the leaf of *F. crocata* (Ace-EFc). AA: ascorbic acid, was used as standard. IC_{50} of Ace-EFc on DPPH = 107.05 ± 2.61 µg/mL, IC_{50} of AA on DPPH = 118 ± 2.48 µg/mL (p>0.05). IC_{50} of Ace-EFc on ABTS = 1.47 ± 1.21 µg/mL, IC_{50} of AA on ABTS = 2.22 ± 0.80 µg/mL (p<0.05). t student test.
